# An unusual cause of obstructive sleep apnea syndrome

**DOI:** 10.1002/ccr3.2345

**Published:** 2019-07-28

**Authors:** Giovanni Briganti, Jérôme R. Lechien, Ivan Theate, Sven Saussez

**Affiliations:** ^1^ Department of Human Anatomy and Experimental Oncology, School of Medicine, UMONS Research Institute for Health Sciences and Technology University of Mons Mons Belgium; ^2^ Institute of Pathology and Genetics Charleroi Belgium

**Keywords:** apnea, lymphoma, obstructive, sleep, tonsil

## Abstract

The histopathologic examination is important after tonsillectomy, irrespective to the surgical indication.

## 1 INTRODUCTION

The histopathologic examination is still important after tonsillectomy, irrespective to the surgical indication. In this article, we report a case of tonsil malignancy manifested as obstructive apnea syndrome.

Obstructive sleep apnea syndrome (OSA) is defined as the recurrent reduction or cessation of the airflow due to the presence of an obstruction in the upper airway. The prevalence of OSA is 14% and 5% in male and female, respectively.^1^ Enlarged soft tissue structures both within and surrounding the airway significantly contribute to airway narrowing in most cases of OSA.^2^ Many anatomical factors are associated with the development of OSA including obesity, nasal septal deviation, inferior turbinate hypertrophy, crowding of the posterior oropharyngeal wall, retrognathia, or tonsillar hypertrophy. Precisely, tonsillar hypertrophy is one of the most prevalent causes of lateral pharyngeal wall thickening associated with OSA.^3^ However, a recent study showed that the severity of palatine tonsil grade is correlated with the severity of the apnea‐hypopnea index.^4^ These anatomical characteristics lead to the development of a myriad of symptoms, that is, daytime sleepiness, nocturnal waking due to choking, reduced concentration, morning headaches, nocturia, irritability, and erectile dysfunction.^5^ According to the OSA severity and patient compliance, the treatment involves weight loss, continuous positive airway pressure (CPAP), and surgery in the case of noncompliance or resistance to nonsurgical approaches. The most commonly performed surgical procedure for OSA is uvulopalatopharyngoplasty with or without tonsillectomy. In this paper, we report an unusual cause of OSA in a patient with a major tonsillar hypertrophy.

## 2 CASE HISTORY

**Figure 1:**
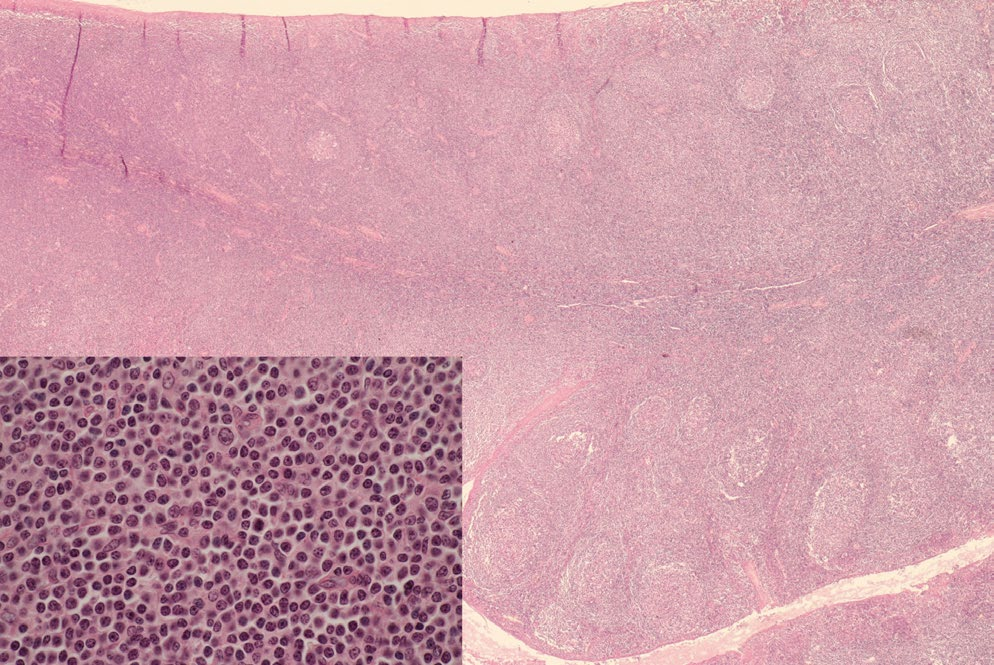
Low‐power and high‐power examination of the tonsils. Low‐power examination of the tonsils showed a relative preservation of the architecture with hyperplasic germinal centers but also a massive expansion of the paracortical zone (HE, Original magnification ×25). High‐power view of this zone displayed diffuse and monotonous infiltration by small round cells containing mottled chromatin. Only rare large cells with nucleolus (paraimmunoblasts) were seen (inset, HE, OMx600)

**Figure 2:**
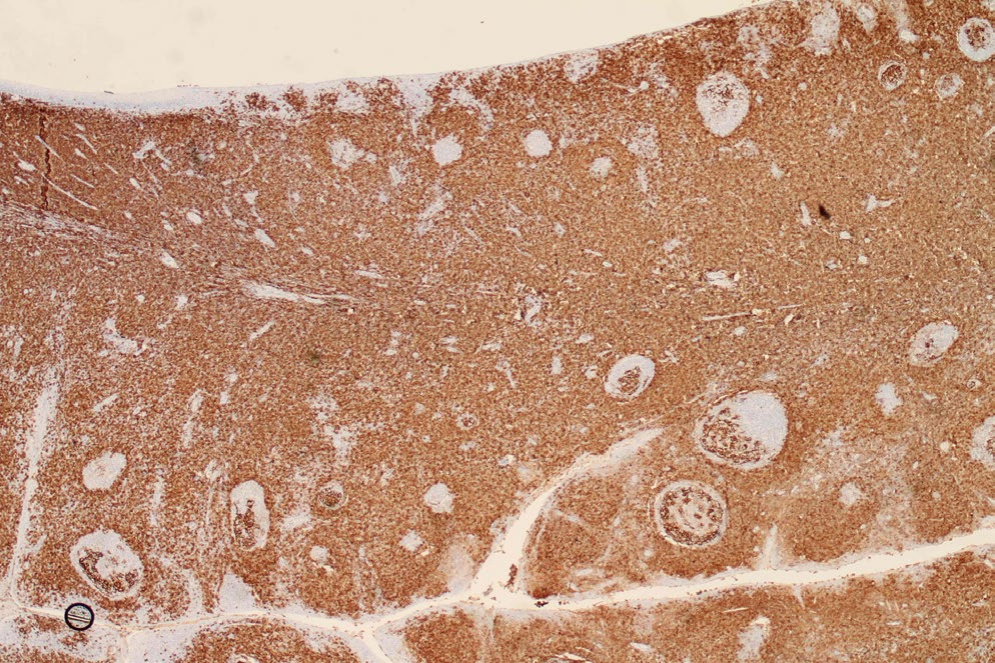
Immunohistochemistry. Paracortical area contained massive infiltration by cells massively positive for CD20, with co‐expression of CD5, CD23, and LEF‐1. Original magnification ×25 and ×400

A 54‐year‐old male was referred to the ear, nose, and throat (ENT) department because of a 12‐month history of snoring, nocturnal waking, and chronic mouth breathing. The ENT clinical examination revealed a grade IV tonsillar hypertrophy, a major septal deviation, and a bilateral inferior turbinate hypertrophy. The nasofibroscopic examination as well as the Muller maneuver suggested that the tonsils were the main anatomical obstructive area during breathing. The rest of the physical examination was unremarkable, and the patient did not have allergies. Polysomnographic evaluation showed light snore, with an apnea‐hypopnea index of 15/h indicating moderate obstructive sleep apnea. According to the anatomical findings, a surgery (uvulopalatopharyngoplasty and tonsillectomy) was proposed and accepted by the patient. The preoperative blood tests were within normal ranges, including normal lymphocytosis. The patient underwent septoplasty, bilateral turbinate radiofrequency, and tonsillectomy. The macroscopic examination of the tonsils showed a 4 × 3.3 × 2.7 cm left tonsil weighted 5 g and a 6 × 4.4 × 2.9 cm right tonsil weighted 9 g. Both specimens showed an elastic consistency at section. The histopathologic examination of the tonsils reported hyperplasia of the germinal centers surrounded by an expanding paracortical area (Figure 1). Immunochemistry showed diffuse infiltration of paracortex by monotonous B cells expressing CD20 with co‐expression of CD5, CD23, and LEF‐1 (Figure 2). Molecular cytogenetic analysis (fluorescent in situ hybridization) reported trisomy 3 in 30% of the nuclei, deletion of 13q (locus D13S319) in 60% of the nuclei, absence of Epstein‐Barr Virus (EBV), and no translocation of MALT1. These analyses conclude to lymphocytic lymphoma/LLC‐B without signs of blastic transformation. The patient recovered normally after surgery and was addressed to the department of hematology.

## 3 DISCUSSION

Tonsillar hypertrophy and related OSA is an extremely rare presentation of lymphoma since only two cases have been reported in the literature, both in children.^6^, ^7^ Lymphomas account for around 14% of malignant tumors of the palatine tonsils, and patient usually has hypertrophy of the Waldeyer ring and/or cervical lymphadenopathy.^8^ However, a recent review of 54 901 patients showed a 0.087% incidence of tonsillar malignancy and only 0.011% of cases received an entirely unsuspected malignant diagnosis,^9^ making a de novo lymphoma diagnosis in an adult patient without specific symptoms extremely rare. Clinical risk factors for malignancy include tonsillar asymmetry, palpable firmness, visible lesions, neck adenopathy, and constitutional symptoms.^10^ Systemic findings as fever, night sweats, and weight loss are not a common feature^11^ and were not present in our case. Our patient only presented sleep disorders that were compatible with OSA and a slight enlargement of palatine tonsil with a slowly evolving tonsillar hypertrophy. This clinical presentation of lymphoma remains exceptional.

Moreover, this case raises the important question of the systematic histopathologic analysis of resected specimens. Indeed, a current and controversial debate exists about the usefulness systematic histopathologic examination in routine tonsillectomy specimens. Some authors state that routine histologic examination is important because it allows the detection of unexpected malignant findings while other suggest that this should only be indicated in case of malignancy suspicion and when risk factors are present.^9^ This case report strengthens the arguments of physicians who support the importance of a pathology review even in normal‐appearing specimens. They support this systematic analysis according to the multiple malignancy cases discovered incidentally without significant risk factors.^12^ Our findings further support the latter statement in favor of the systematic histologic examination. Should some of the palatine tonsillar specimens not be submitted to the laboratory of pathology or only be grossly examined, some malignancies may not be diagnosed in both adult and pediatric patients,^12^ which might lead to delayed management of malignancy and complex medicolegal implications.

## CONFLICT OF INTEREST

None declared.

## AUTHOR CONTRIBUTIONS

GB: wrote the manuscript. JRL: reviewed the manuscript. IT: performed the histologic examination and commented the figures. SS: reviewed the manuscript.
